# Assessment of face mask use in peripartum women during the COVID-19 pandemic: an observational study

**DOI:** 10.1186/s12884-025-07734-6

**Published:** 2025-08-01

**Authors:** Samuel Adusei, Tracey Adams, Chantal Stewart

**Affiliations:** 1 Department of Obstetrics & Gynaecology, Mowbray Maternity Hospital, University of Cape Town, 12 Hornsey Rd, Mowbray, Cape Town, 7700 South Africa; 2 Department of Obstetrics & Gynaecology, Groote Schuur Hospital, University of Cape Town, Cape Town, South Africa; 3 South African Medical Research Council, University of Cape Town Gynaecological Cancer Research Centre (SA MRC UCT GCRC), Cape Town, South Africa

**Keywords:** COVID-19, Peripartum women, Face mask use, Effective use, Knowledge

## Abstract

**Background:**

The new coronavirus disease 2019 (COVID-19) pandemic resulted in significant mortality, particularly in vulnerable groups, including pregnant women. Unavailability of a vaccine in South Africa until February 2021 and slow rollout made it important for the prevention of transmission to rely on non-pharmaceutical interventions such as face mask use. We aimed to assess the rate of face mask use in peripartum women in a secondary hospital in South Africa and to determine knowledge of and reasons for use and non-use of face masks.

**Methods:**

This was a cross-sectional quantitative study using opportunistic sampling of women in the first and second stages of labour and the first six hours postpartum to assess the proportion of women who used face masks effectively between 1 and 31 October, 2020. A smaller sample of women answered a structured interviewer-administered questionnaire to assess their knowledge of face mask use and reasons for wearing or not wearing the face mask. The questionnaire was based on the Health Belief Model.

**Results:**

The study included 500 women. Of those who wore face masks, 81.7% wore them correctly and 18.3% ineffectively. 78% of respondents were aware of how to effectively use a face mask. Rates of effective face mask use were 83.1%, 64.3%, and 81.1% in the postpartum period, second and first stages of labour, respectively. 90% of participants had adequate knowledge of face mask use and this positively correlated with effective face mask wearing. More than half of the respondents, (53.2%), used face masks because they “felt susceptible to getting COVID-19 in the hospital”. The majority felt that their reason for wearing a face mask was to prevent transmission to loved ones (90.8%) or to protect themselves (96%). More than half of the women, (54.0%), did not find face mask wearing troublesome. Face mask use was lowest in the second/third stages of labour (*p* = 0.016) and in women with secondary rather than tertiary education (*p* = 0.016).

**Conclusion:**

Information on non-pharmaceutical interventions during the COVID-19 pandemic in a low- middle income country will be useful to inform educational and other strategies in future respiratory virus outbreaks.

**Supplementary Information:**

The online version contains supplementary material available at 10.1186/s12884-025-07734-6.

## Background

In the past decades there have been several serious outbreaks of respiratory viral illness which have caused significant mortality across the world. These have included severe acute respiratory syndrome (SARS) in 2003, Middle Eastern respiratory syndrome in 2012, and H1N1 and H5N1 in 2014 [1]. The onset of the severe acute respiratory syndrome coronavirus 2 (SARS-CoV-2) pandemic which began in Wuhan, China in 2019 and was declared a public health emergency by the World Health Organization (WHO) in 2020 [2], demonstrated the severe effect that widespread viral infections can have on individuals, the world, and society.

Coronavirus disease (COVID-19) has a high morbidity and infectivity rate, particularly in the elderly and those with comorbidities. Pregnant women are particularly vulnerable because of altered immunity during pregnancy. While they are not more susceptible to infection, they are at higher risk of severe symptoms and complications, including pneumonia, acute adult respiratory distress syndrome and cardiac injury [3–5].

A systematic review and meta-analysis including 74 studies from various geographic regions aiming to assess the impact of COVID-19 on maternal and neonatal outcomes identified an increase in the pooled prevalence of preterm delivery, maternal mortality, neonatal intensive care unit (NICU) admission, and neonatal death [6]. Furthermore, the prevalence of pregnancy loss and SARS-CoV-2 positive neonates were higher in low- to-middle income countries (LMIC) than in higher income countries.

One of the major factors in the rapid spread of SARS-CoV-2 was its high airborne transmissibility. Owing to this, governments in many countries imposed measures to decrease transmission. At the beginning of the pandemic, specific vaccine trials were still ongoing and, later, implementation of mass vaccination programmes proved challenging, particularly in LMIC countries. Thus, non-pharmaceutical measures, including social distancing, frequent hand washing, and wearing of face masks were introduced at a population level as recommended by the WHO and Centers for Disease Control and Prevention (CDC) [7, 8]. South Africa made the wearing of face masks in public compulsory from 1 May 2020. A strong public media campaign resulted in high acceptance rates.

During labour and delivery women often forcibly exhale, and birth attendants may thus be exposed to droplets containing virus, predisposing them to an additional risk of viral transmission. They are also at risk from exposure to vaginal secretions, necessitating the wearing of personal protective equipment (PPE). Compliance with face mask wearing has been assessed in studies outside the hospital environment. Previous studies during other respiratory virus outbreaks found a willingness to comply with face mask wearing in only 4–50% of cases [9–11]. This highlights a need to investigate the determinants of face mask-wearing, identify the issues and overcome the barriers associated with face mask-wearing compliance.

We thus aimed to determine the proportion of peripartum women who effectively used face masks during labour and the immediate postpartum period, as well as to elucidate the reasons for use or non-use.

## Methods

### Study design

This was a cross-sectional quantitative study using opportunistic sampling of women in the first and second stages of labour and the first six hours postpartum at Mowbray Maternity Hospital to assess the proportion of women using face masks correctly, incorrectly, or not at all between 1 October 2020 and 31 October 2020. A smaller sample of women answered a structured questionnaire assessing their knowledge of and reasons for correct, incorrect, or no use of face masks.

### Patients and study site

Mowbray Maternity Hospital is a secondary level referral hospital in the Metro West area of the Western Cape, South Africa which serves the largely indigent population and performs approximately 10 000 deliveries per year.

Women were included in the study if they were in the first or second stages of labour or the first six hours postpartum, were COVID-negative, and had no contraindications to face mask use.

Exclusion criteria were contraindications to face mask use, such as severe acute asthma, dyspnoea or allergy.

The first stage of labour was defined as onset of contractions with cervical change up to a dilatation of 10 cm. The second stage of labour was defined as from 10 cm dilatation to delivery of the baby. The immediate postpartum period was defined as from delivery of the baby to 6 h postpartum.

### Methodology

#### Sampling

After sample size calculation, simple random sampling was used to select study participants from each stage of labour for inclusion. This was done by assigning numbers to the beds and including women occupying those beds each day (Supplementary file 1).

### Observation

First, the investigator observed women in each stage of labour over a 10-minute period, using a check list to record face mask usage data. Effective face mask usage was assessed using five items adapted from the WHO guidelines on face mask usage [2]: coloured side of the face mask should face outwards; metallic strip should be on the upper side; the elastic band should be firmly positioned; the face mask should be extended to cover the mouth, nose and chin; the face mask should not be touched once secured. Anyone who had all five items correct was deemed to have effectively worn their face mask.

The type of face mask (cloth, surgical or N95) was recorded as well as maternal demographic data, including age, educational level, marital status, household income, and employment status.

### Questionnaire

A subgroup of women was then selected to answer a structured interviewer-administered questionnaire to assess pregnant women’s knowledge of face mask use and reasons for wearing or not wearing the face mask. The questionnaire was based on the Health Belief Model which suggests that prevention is due to the extent to which people interpret a personal health threat and their confidence that certain preventive activities will minimise the threat [12]. In applying this model to understand the practice of COVID-19 preventive behaviours, perceived health threat refers to individuals’ perception of how vulnerable they are to contracting COVID 19 (***perceived susceptibility***: demographic characteristics, knowledge, and awareness of local outbreaks) and that this disease has serious consequences (***perceived severity***: including a personal history of infection, contact history, knowledge of fatality rate of disease, degree of concern for family). Individuals’ belief that the practice of face mask wearing will prevent COVID-19 depends on whether they think these preventive behaviours will be effective (***perceived benefits***), and whether the cost of undertaking these behaviours exceeds the disadvantages (***perceived barriers***, including discomfort, inconvenience, difficulty with respiration and allergy), and whether there are any cues (**cues to action**) to encourage these behaviours, such as understanding of one’s state of body, or external cues such as the effect of social media and social pressure.

Eight items were generated to assess knowledge of effective face mask use (1 point for each correct answer, 0 points for incorrect answers). The knowledge score was obtained by summation of responses to each question. Participants were classified into Group 1 (correct use/adequate knowledge) if they scored ≥ 50% and Group 2 (incorrect use/inadequate knowledge) if they scored < 50% on the questions.

### Data collection

Direct observation was carried out by SA or a suitably trained medical officer and recorded on the observation check list. Questionnaires were administered by the same individuals. The observation check lists and questionnaires were anonymised and entered into a spreadsheet for further analysis.

### Statistics

STATA version 15 (StataCorp LLC, Texas, USA) was used for statistical analysis. Frequencies and percentages were reported for categorical variables. Means and standard deviation were determined for continuous variables. Graphs and percentages were used to report the proportion of women who effectively used face masks in each stage of labour. Additionally, the chi-square or Fisher’s exact test was used to determine a statistically significant association between the dependent variable (effective face mask use) and each independent variable. Bivariate analysis was used to determine both the association and the strength of association with the outcome variable. Factors identified as statistically significant after bivariate analysis were then fitted into a multiple logistic regression model to determine the factors associated with face mask use. Crude and adjusted odds rations were reported with 95% confidence intervals and statistical significance was set at *p* < 0.05.

### Ethics

All women signed informed consent and the study was approved by the Ethics Committee of the University of Cape Town (HREC 832/2020). The study was conducted in accordance with the guidelines laid out in the Declaration of Helsinki (amended 2013).

## Results

A total of 500 women were included in the study. Of these, 476 (95.2%) were observed to be wearing a face mask. Of those who wore face masks, 389 (81.7%) wore it correctly and 87 (18.3%) ineffectively.

From this group, 250 women answered questions to determine their knowledge of effective face mask use (Table 1).

**Table 1:** Technique of using a face mask

	Correct *n* (%)	Incorrect *n* (%)
Ensure the coloured side of the face mask is facing outwards	227 (90.8)	23 (9.2)
Ensure the metallic strip is on the upper side	224 (89.6)	26 (10.4)
Position the elastic band correctly	228 (91.2)	22 (8.8)
Extend the face mask to cover mouth, nose, and chin	218 (87.2)	32 (12.8)
Avoid touching the face mask once it is secured	219 (87.6)	31 (12.4)

**Fig. 1:**
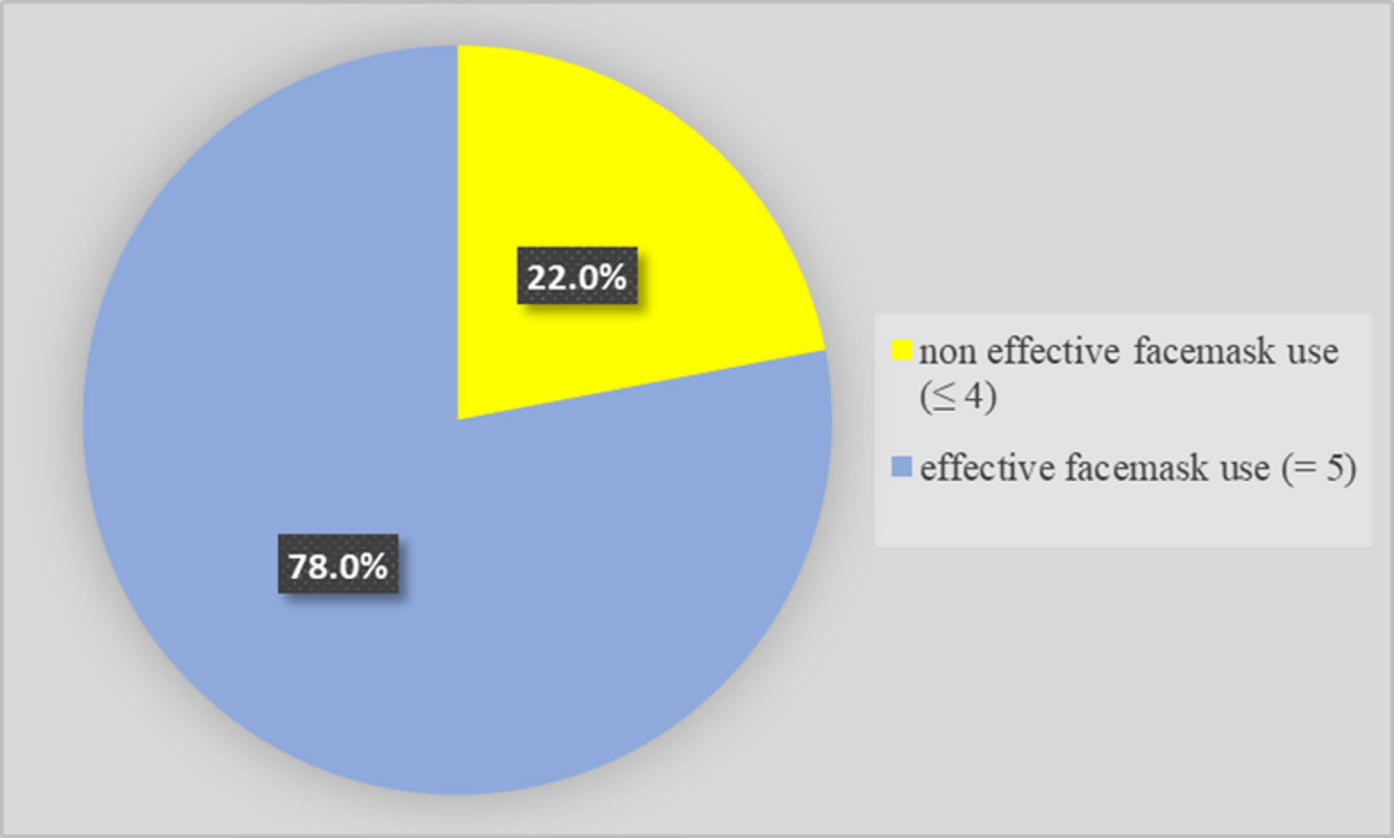
Effective face mask usage

Of the respondents, 78% were aware of how to effectively use a face mask (Fig. 1). Table 2 shows the demographic details of the women and the stage of labour when they were recruited.

**Table 2:** Sociodemographic characteristics of respondents (*n* = 250)

Variables	Total *n* (%)	First stage *n* (%)	Second stage *n* (%)	Post-partum stage *n* (%)
**Age distribution**				
18–20 years	19(7.6)	12(10.8)	1(1.8)	6(7.2)
21–30 years	134(53.6)	53(47.8)	36(64.2)	45(54.2)
31–40 years	86(34.4)	42(37.8)	17(30.4)	27(32.5)
Over 40 years	11(4.4)	4(3.6)	2(3.6)	5(6.0)
**Parity**				
Primigravid	96(38.4)	48(43.2)	20(35.7)	28(33.7)
Multigravida	154(61.6)	63(56.8)	36(64.3)	55(66.3)
**Relationship status**				
Single	96(38.4)	43(38.7)	19(33.9)	34(41.0)
Married	138(55.2)	57(51.4)	35(62.5)	46(55.4)
Cohabiting	12(4.8)	7(6.3)	2(3.6)	3(3.6)
Divorced	4(1.6)	4(3.6)	0(0.0)	0(0.0)
**Educational level**				
None	2(0.8)	1(0.9)	1(1.8)	0(0.0)
Primary	6(2.4)	2(1.8)	2(3.6)	2(2.4)
Secondary	192(76.8)	83(74.8)	46(82.1)	63(75.9)
Tertiary	50(20.0)	25(22.5)	7(12.5)	18(21.7)
**Employment status**				
Employed	97(38.8)	39(35.1)	21(37.5)	37(44.6)
Unemployed	153(61.2)	72(64.9)	35(62.5)	46(55.4)
**Allergies**				
No	244(97.6)	109(98.2)	54(96.4)	81(97.6)
Yes	6(2.4)	2(1.8)	2(3.6)	2(2.4)

**Fig. 2:**
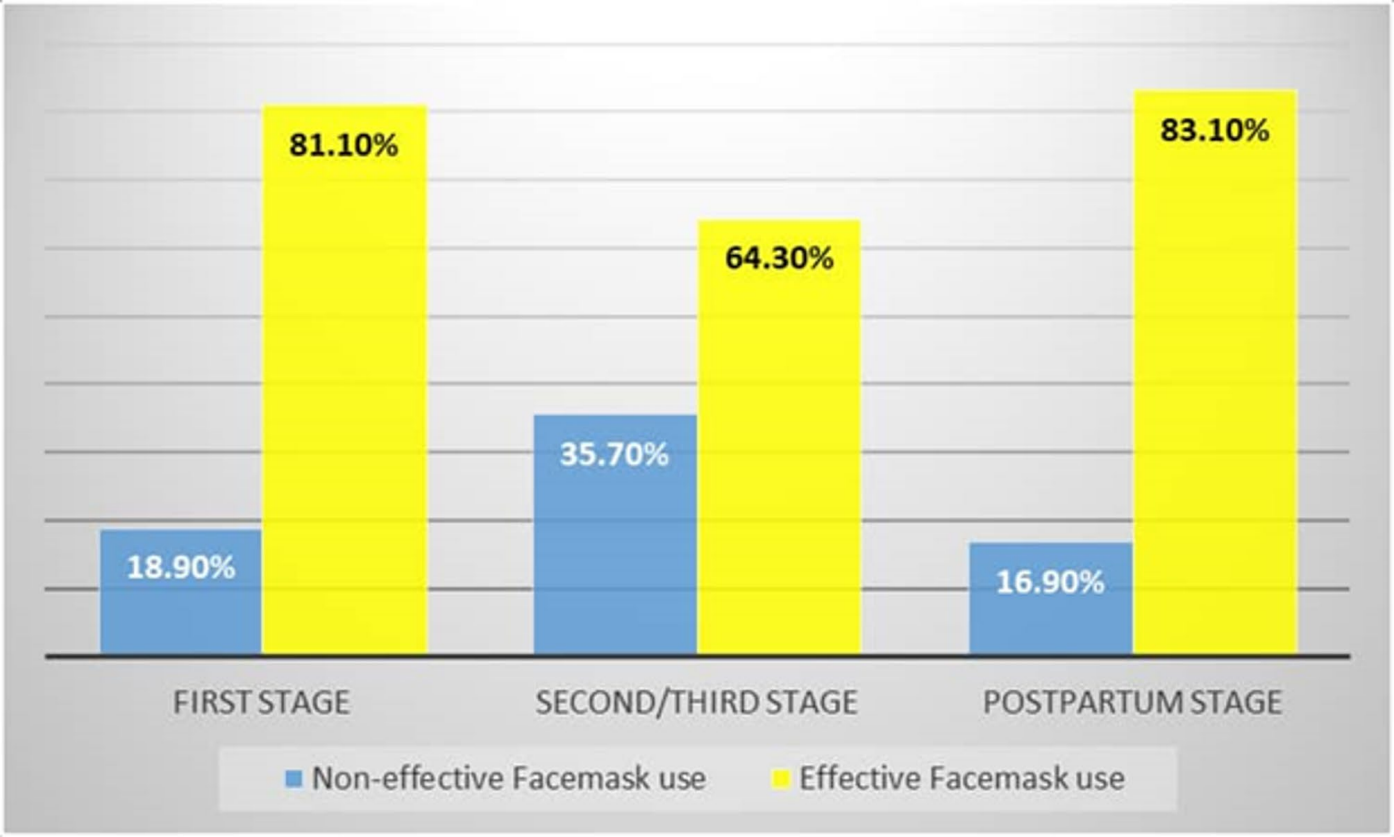
Effective face mask use by stage of labour *P* = 0.018

Effective face mask use was highest in the postpartum period (83.1%). The second stage of labour recorded the lowest prevalence (64.3%) of effective face mask use as compared to the first stage of labour (81.1%) and the postpartum period (83.1%) (Fig. 2). *p* = 0.018 (statistically significant).

We then went on to assess women’s knowledge of face mask use (Table 3) and found that 90% had adequate knowledge.

**Table 3:** Knowledge of face mask use

Variables	True *n* (%)	False *n* (%)	Don’t know *n* (%)
**When wearing a face mask at the clinic**,** there** **is no need to cover the mouth when sneezing or** **coughing**	89(35.6)	160(64.0)	1(0.4)
**A cloth mask is as effective as a regular surgical face mask**	151(6.4)	76(30.4)	23(9.2)
**The used face mask can be stored for later use when** **one is not sick**	57(22.8)	187(74.8)	6(2.4)
**A face mask helps to prevent HIV**	13(5.2)	232(92.8)	5(2.0)
**Perform hand hygiene before face mask wearing**	232(92.8)	13(5.2)	5(2.0)
**Choose the appropriate size of face mask**	209(83.6)	35(14.0)	6(2.4)
**Dispose of the used face mask in a lidded rubbish bin**	233(93.2)	13(5.2)	4(1.6)
**Touch only the elastic band when removing the face mask**	222(88.8)	16(6.4)	12(4.8)

We assessed reasons for use/non-use of face masks. More than half of the respondents, 133 (53.2%) agreed that they used face masks because they “felt susceptible to getting COVID-19 in the hospital”. The majority of the respondents, 227 (90.8%) agreed that “having COVID-19 will be troublesome as it may spread to loved ones” as a reason for using face masks. 96% (240/250) agreed that they used face masks due to the belief that wearing a face mask is a good way to protect oneself and others against COVID-19 in the hospital. More than half of respondents, 135 (54.0%) disagreed that “wearing a face mask is troublesome because it distorts communication” while 138 (55.2%) agreed that they “sometimes forgot to put on their face mask”. 56% (140/250) of the respondents agreed that their reason for using a face mask was only if the doctor/nurse said so.

We explored the association between effective face mask use and reasons for use or non-use of face masks (Supplementary Table 1). There was a statistically significant association between respondents’ belief that having COVID-19 would be troublesome as it might lead to taking time off work (*p* = 0.010) and pressure from mass media and government reminding them of the need to put on face mask (*p* = 0.005), and effective face mask use.

In terms of sociodemographic factors associated with effective face mask use, the only factor which showed a statistically significant association was the educational level **(***p* = 0.015**)**.

The results of the bivariate analysis (age distribution, parity, relationship status, educational level, employment status, and allergies) are shown in Supplementary Table 2.

The results of multiple logistic regression analysis of factors associated with effective face mask use are shown in Table 4. Respondents in the second/third stage of labour had a 67% reduction in their odds of effective face mask use compared to those in the first stage of labour (crude odds ration [cOR] = 0.33; 95% confidence intervals [CI] = 0.16–0.68; *p* = 0.003). However, after adjusting for all other factors, the reduction in effective face mask use was 62% among respondents in the second/third stage of labour compared to those in the first stage of labour (adjusted odds ratio [aOR] = 0.38; 95% CI = 0.17–0.83; *p* = 0.016).

**Table 4:** Factors associated with effective face mask use

Variables	cOR (95% CI)	*p*-value	aOR (95% CI)	*p*-value
Labour stage				
**First stage**				
Final stage	**0.33(0.16–0.68)**	0.003*	**0.38(0.17–0.83)**	0.016*
Postpartum stage	1.53(0.69–3.37)	0.295	1.74(0.70–4.30)	0.233
**Educational level**				
Tertiary	1.00		1.00	
None	0.09(0.005–1.67)	0.105	0.05(0.002–1.20)	0.065
Primary	0.43(0.04–4.69)	0.492	0.33(0.03–4.18)	0.393
Secondary	**0.25(0.09–0.74)**	0.012*	**0.25(0.08–0.77)**	0.016*
**Having COVID-19 will be troublesome as it may lead to taking time off work**				
Agree	1.00		1.00	
Uncertain	**0.26(0.09–0.71)**	0.008*	0.34(0.12–1.02)	0.054
Disagree	2.02(0.58–7.05)	0.269	2.34(0.60–9.08)	0.221
**Pressure from mass media and government reminds one of need to put on face mask**				
Agree	1.00		1.00	
Uncertain	**2.33(1.04–5.22)**	0.040*	**2.66(1.08–6.51)**	0.033*
Disagree	**3.28(1.43–7.52)**	0.005*	**3.58(1.44–8.93)**	0.006*
**Overall knowledge**				
Inadequate knowledge	1.00		1.00	
Adequate knowledge	**2.67(1.12–6.33)**	0.026*	**4.10(1.49–11.28)**	0.006*

OR, odds ratio; CI, confidence intervals

There was a 75% reduction in effective face mask use among respondents with up to secondary as compared to tertiary school education (cOR = 0.25; 95% CI = 0.09–0.74; *p* = 0.012), while adjusting for other factors (labour stage, pressure from mass media, knowledge of face mask use) did not change this (aOR = 0.25; 95% CI = 0.08–0.77; *p* = 0.016).

Respondents who were uncertain whether “having COVID-19 will be troublesome as it may lead to taking time off work” had a 74% reduction in their odds of effective face mask use compared to those who agreed with the statement. However, after adjusting for other factors, this association was no longer statistically significant.

Effective face mask use was significantly increased among uncertain respondents (cOR = 2.33; 95% CI = 1.04–5.22; *p* = 0.040) and those who disagreed (cOR = 3.36; 95% CI = 1.41–8.01; *p* = 0.005) with the statement that “pressure from mass media and government reminded them of the need to put on a face mask”. After adjusting for all other factors, this significant increase was still present among respondents who were uncertain (aOR = 2.66; 95% CI = 1.08–6.51; *p* = 0.033) or disagreed with the statement (aOR = 3.58; 95% CI = 1.44–8.93; *p* = 0.006).

Furthermore, having adequate knowledge of face mask use significantly increased the odds of effective use by 2.67 fold (cOR = 2.67; 95% CI = 1.12–6.33; *p* = 0.026). This increase was greater after adjusting for all other factors (aOR = 4.10; 95% CI = 1.49–11.28; *p* = 0.006).

## Discussion

There have been several respiratory virus epidemics in the last few decades, of which the COVID-19 pandemic was the most widespread and resulted in high mortality; it is likely that more will occur in the future. In most cases, specific vaccines are not available initially and reliance is placed on non-pharmaceutical measures. Vulnerable groups, of which pregnant women are one, are particularly at risk.

According to a population-based cohort study among 36 hospitals in South Africa that assessed pregnancy outcomes of hospitalised pregnant women with COVID-19, of the 673 infected hospitalised pregnant women, 32.2% were admitted for COVID-19 illness and for other indications, there were 39 deaths, 179 (35.4%) preterm births, and 25 (4.7%) stillbirths [13]. It is thus important to determine the uptake of these non-pharmaceutical interventions. Such information will inform the response to future outbreaks of severe respiratory viral illness.

The Royal College of Midwives recommendations suggest that women in labour should not be expected to wear face masks [14]. However, in LMIC countries where vaccine rollout and access is problematic even when a vaccine is available globally, this may be an important method of preventing transmission.

In our study, 78% of peripartum women used face masks effectively during the COVID-19 pandemic. Furthermore, 90% of respondents had adequate knowledge of face mask use and this knowledge was a significant predictor of correct use of face masks (*p* = 0.006). In exploring the reasons for effective face mask use, more than half the women used face masks because they felt susceptible to getting COVID-19 in hospital and the majority used face masks because they believed that they were effective in preventing COVID-19 and decreasing its spread to loved ones. The lowest rate of face mask usage was in the second stage of labour. Higher education levels were associated with a higher rate of face mask wearing.

The rate of face mask use in the peripartum period is relatively high, considering that the pain, energy expenditure, and emotion of labour was thought to be a possible deterrent to effective face mask use. Studies have shown rates of face mask use varying from 91.8% in an outpatient setting to 4% in a community setting during the SARS pandemic [15, 16]. The relatively good results in our study may relate to the fact that the study was conducted in the hospital at a time when there was widespread information about the need to wear face masks, coupled with the country’s directive for individuals to adhere to COVID-19 protocols.

The rate of knowledge of effective face mask use of 90% and the consequent association between knowledge and adequate face mask usage (4-fold increase; *p* = 0.006) concurs with the findings of a questionnaire-based cross-sectional study conducted in a primary health care facility in South Africa by Hoque et al. assessing knowledge, attitude and practices among pregnant women during the COVID-19 pandemic [17]. They showed that women with good knowledge were 7 times more likely to practice positively regarding COVID-19 (*p* = 0.019). A Kenyan study, however, showed that, while knowledge of the advantages of non-pharmaceutical interventions was high amongst a group of pregnant women (99%), this did not translate to good practice [18]. The reasons for non-use of face masks was that they were uncomfortable and expensive. In our study, face masks were made available free to all women attending the hospital. In contrast, research conducted among healthcare workers in Pakistan in April 2020 to assess their knowledge, attitude, and practices of face mask use in limiting the spread of COVID-19 revealed inadequate knowledge of the technique and practice of face mask use. Of 392 participants, only 43.6% knew how to wear the face masks properly, 68.9% recognised that there were three layers, and 75.5% knew the prescribed maximum wearing period [19]. Our study was conducted slightly later in the pandemic which may explain the difference as there would have been more time to disseminate information. A recent meta-analysis of knowledge, attitudes and practice of pregnant women in Africa regarding COVID-19 [20] reported a knowledge rate of 61.8% and positive preventative practices in 52.3% of women. The studies included were mainly from the Eastern and Western regions of Africa, with only one study [17] from southern Africa. These results also reflect regional differences with higher knowledge in the Western region. They recommend health education in antenatal clinics. In our setting, health education on COVID-19 was routinely made available during antenatal visits, sensitising women on the need for COVID prevention and face mask wearing.

In exploring women’s reasons for wearing face masks, more than half felt susceptible to contracting COVID-19 in hospital and the majority believed that COVID-19 was troublesome and could spread to loved ones and that wearing face masks was a way of preventing this (90.8% and 96%, respectively). The majority did not find it troublesome to wear a face mask and those who effectively wore their face masks did not do so because of pressure from mass media, but rather because of their knowledge and beliefs about the benefits of face mask use.

As in other studies, we found that face mask usage was greater in women with higher levels of education [21–23]. There is a need to make educational materials on effective face mask use and COVID-19 simple through public education and outreach programs to target this subgroup of the population with lower education levels. Consideration should be given to the use of easily accessible means such as television and addressing potential discomfort.

Effective face mask use was highest during the postpartum stage (83.1%) and lowest in the second stage of labour (64.3%) with the first stage of labour recording 81.1%. The relatively low prevalence in the second stage of labour is thought to be due to the pain and emotion that characterises this stage of labour and the potential discomfort that the face mask use may pose to the respondents, as the women are pushing and breathing heavily, and this can be hampered by wearing a face mask. However, this finding is significant because of the potential for the spread of COVID-19 at this stage of labour.

Consideration needs to be given to the high risk of SARS-CoV-2 transmission to delivery assistants and birth companions at the second stage of labour, emphasising the importance of appropriate PPE.

This study has some limitations. It was a single centre study and thus might not be generalisable. It is likely that similar results would be obtained in large urban hospitals but further research should be conducted in rural areas.

Based on the cross-sectional study design, associations found cannot be temporal or causal.

The observation of effective face mask use can be prone to observer bias. However, the robust statistical methods used in the conduct of the study make the findings empirically sound and can be compared to findings from similar contexts.

## Conclusion

The rate of effective face mask use was high amongst respondents but was lowest in the second stage of labour. Knowledge of effective face mask use was also high.

Perceived susceptibility to getting COVID-19 in the hospital and the potential for transmission to families were reasons why most respondents wore face masks effectively.

Women in the second of labour had the lowest proportion of effective face mask use as did those with secondary rather than tertiary education. This information may be of value in future outbreaks of respiratory viral diseases and can inform education and outreach programs promoting non-pharmaceutical interventions. These should focus on ongoing antenatal education, outreach programs to low-literacy populations, and more effective use of social media platforms to disseminate information.

## Electronic supplementary material

Below is the link to the electronic supplementary material.

Supplementary Material 1

Supplementary Material 2

Supplementary Material 3

## Data Availability

Data is available from Dr Samuel Adusei on reasonable request.

## Abbreviations

aOR: Adjusted odds ratio
CDC: Centers for Disease Prevention
CI: Confidence interval
cOR: Crude odds ratio
COVID-19: New coronavirus disease 2019
LMIC: Low- middle income countries
OR: Odds ratio
PPE: Personal protective equipment
SARS: Severe acute respiratory syndrome
SARS-CoV-2: SARS coronavirus 2
WHO: World Health Organization
