# Supplementary material for: Assessment of face mask use in peripartum women during the COVID-19 pandemic: an observational study
Source: BMC Pregnancy Childbirth. 2025 Aug 1;25:804. doi: 10.1186/s12884-025-07734-6 (PMC12317551; doi:10.1186/s12884-025-07734-6)
Supplement: Supplementary file 1 — Supplementary Material 1 [file 12884_2025_7734_MOESM1_ESM.docx]

Supplementary Table 1. Association between effective use of facemask and reasons for use/non-use of facemask

| Variables | Effective facemask use | | p-value |
| --- | --- | --- | --- |
|  | Yes  (n = 195) | No  (n = 55) |  |
| Feeling susceptible to getting COVID in the hospital |  |  | 0.785 |
| Agree | 105(78.9) | 28(21.1) |  |
|  |  |  |  |
| Belief there is a high chance of having COVID transmitted whilst at the hospital |  |  | 0.481 |
| Agree | 97(75.2) | 32(24.8) |  |
|  |  |  |  |
| Feeling that since COVID crisis is over, there is no worry about getting COVID-19 |  |  | 0.523 |
| Agree | 49(79.0) | 13(21.0) |  |
|  |  |  |  |
| Belief that getting COVID-19 is serious |  |  | **^+^**0.107 |
| Agree | 176(78.9) | 47(21.1) |  |
|  |  |  |  |
| Having COVID-19 will be troublesome as it may spread to loved ones |  |  | **^+^**0.760 |
| Agree | 177(78.0) | 50(22.0) |  |
|  |  |  |  |
| **Having COVID-19 will be troublesome as it may lead to taking time off work** |  |  | **^+^0.010*** |
| Agree | 163(79.1) | 43(20.9) |  |
|  |  |  |  |
| Belief that wearing a face mask is a good way to protect oneself and others against COVID in the hospital |  |  | **^+^**0.482 |
| Agree | 186(77.5) | 54(22.5) |  |
|  |  |  |  |
| Wearing a facemask in the hospital cannot prevent transmission of COVID-19 |  |  | **^+^**0.946 |
| Agree | 113(78.5) | 31(21.5) |  |
|  |  |  |  |
| Will only wear a facemask in the hospital if it is free |  |  | **^+^**0.146 |
| Agree | 36(69.2) | 16(30.8) |  |
|  |  |  |  |
| Wearing a facemask is troublesome because distorts communication |  |  | **^+^**0.331 |
| Agree | 69(74.2) | 24(25.8) |  |
|  |  |  |  |
| Wearing a facemask is troublesome because it makes breathing difficult when put on |  |  | **^+^**0.311 |
| Agree | 84(75.7) | 27(24.3) |  |
| Sometimes forget to put on the mask |  |  | 0.439 |
| Agree | 104(75.4) | 34(24.6) |  |
|  |  |  |  |
| Will feel shame as the only person wearing a facemask in the hospital |  |  | 0.331 |
| Agree | 51(76.1) | 16(23.9) |  |
|  |  |  |  |
| Will wear a facemask if there were more posters serving as a reminder |  |  | 0.100 |
| Agree | 74(71.8) | 29(28.2) |  |
|  |  |  |  |
| Will wear a facemask if the doctor/nurse say so |  |  | 0.425 |
| Agree | 105(75.0) | 35(25.0) |  |
|  |  |  |  |
| Pressure from employers at work force one to put on mask |  |  | 0.506 |
| Agree | 84(75.0) | 28(25.0) |  |
|  |  |  |  |
| **Pressure from mass media and government reminds one of need to put on facemask** |  |  | **0.005*** |
| Agree | 87(69.6) | 38(30.4) |  |

­­­­­­­­­­­­­­­­­­­­­­­­_____________________________________________________________________________________
