# Supplementary material for: Assessment of face mask use in peripartum women during the COVID-19 pandemic: an observational study
Source: BMC Pregnancy Childbirth. 2025 Aug 1;25:804. doi: 10.1186/s12884-025-07734-6 (PMC12317551; doi:10.1186/s12884-025-07734-6)
Supplement: Supplementary file 2 — Supplementary Material 2 [file 12884_2025_7734_MOESM2_ESM.docx]

| Supplementary Table 2 Association between socio-demographic characteristics and effective facemask use | | | |
| --- | --- | --- | --- |
| **Variables** | Effective facemask use | | p-value |
|  | Yes  (n = 195) | No  (n = 55) |  |
| **Age distribution** |  |  | **^+^**0.090 |
| 18-20 years | 15(78.9) | 4(21.1) |  |
| 21-30 years | 111(82.8) | 23(17.2) |  |
| 31-40 years | 63(73.3) | 23(26.7) |  |
| Over 40 years | 6(54.5) | 5(45.5) |  |
|  |  |  |  |
| **Parity** |  |  | 0.506 |
| Primigravid | 77(80.2) | 19(19.8) |  |
| Multigravida | 118(76.6) | 36(23.4) |  |
|  |  |  |  |
| **Relationship status** |  |  | **^+^**0.308 |
| Single | 77(80.2) | 19(19.8) |  |
| Married | 108(78.3) | 30(21.7) |  |
| Cohabiting | 8(66.7) | 4(33.3) |  |
| Divorced | 2(50.0) | 2(50.0) |  |
|  |  |  |  |
| **Educational level** |  |  | **^+^0.015*** |
| None | 1(50.0) | 1(50.0) |  |
| Primary | 5(83.3) | 1(16.7) |  |
| Secondary | 143(74.5) | 49(25.5) |  |
| Tertiary | 46(92.0) | 4(8.0) |  |
|  |  |  |  |
| **Employment status** |  |  | 0.094 |
| Employed | 81(83.5) | 16(16.5) |  |
| Unemployed | 114(74.5) | 39(25.5) |  |
|  |  |  |  |
| **Allergies** |  |  | **^+^**0.601 |
| No | 189(79.7) | 48(20.3) |  |
| Yes | 6(100.0) | 0(0.0) |  |
