# Supplementary material for: Assessment of face mask use in peripartum women during the COVID-19 pandemic: an observational study
Source: BMC Pregnancy Childbirth. 2025 Aug 1;25:804. doi: 10.1186/s12884-025-07734-6 (PMC12317551; doi:10.1186/s12884-025-07734-6)
Supplement: Supplementary file 3 — Supplementary Material 3 [file 12884_2025_7734_MOESM3_ESM.docx]

## Supplementary File 1

## Sample Size Determination

To obtain an appropriate sample size for the study, the formula for estimating proportion was used to calculate the sample size. The formula is denoted as follows:

n= N/ (1+N (e) 2)

Where**n**= required sample size, **N**= sample frame (population under study), **e**= margin of error in this case 5%.

The total average vaginal deliveries per month are about 500, thus N is 500. Using the above formula; n= 500/ (1+500(0.05)2) = 223.  **Therefore, a sample size of 223 was used.**

Adjustment for a 10% rate of non-responses of **223** yielded a final sample size of **250.**

The estimated sample size of 250 is expected to include all stages of labour.

The population was sampled from three data collection points. Hence the sample size was trichomised using the beds/unit ratio. The beds/unit ratio for the three data collection points is 8:4:6 for the first stage, labour ward, and immediate postpartum ward, respectively.

The overall sample size divided by this ratio resulted in a sample of (8/18 *250) **111**, (4/18 *250) **56**, and (6/18*250) **83** from the first stage, second stage/ third stage, and immediate postpartum ward respectively.

The total sampling frame of 500 patients were observed using the checklist, from which 250 patients representing the sample size were selected to answer the structured .questionnaire

**Sampling**

**Simple random sampling**was employed to select study participants from each stage to obtain the calculated daily proportionate sample size to answer the structured questionnaire (thus 4 for the first stage, 2-second stage, and 3 immediate postpartum). Beds were numbered and random selection was done. For example, to select 4 patients from the estimated 8 in the first stage, 8 papers were numbered and balloted, those who chose 1 to 4 had their beds numbered and anyone who subsequently occupied those beds was recruited for answering the structured questionnaire unless they did not give consent. This was replicated over the data collection period till the desired sample size for each stage was obtained.
